# Assessment of Flexible Dosing Volumes in an Existing Spring-Driven Autoinjector Platform

**DOI:** 10.3390/pharmaceutics18070818

**Published:** 2026-07-01

**Authors:** Rozhin Derakhshandeh, Javad Eshraghi, Pavlos P. Vlachos, Jean-Christophe Veilleux

**Affiliations:** 1 School of Mechanical Engineering, Purdue University, West Lafayette, IN 47907, USA; rderakhs@purdue.edu; 2 Eli Lilly and Company, Indianapolis, IN 46285, USA; javad.eshraghi@lilly.com (J.E.); jc@alumni.caltech.edu (J.-C.V.)

**Keywords:** subcutaneous injection, drug delivery systems, autoinjector platform, dosing decisions, flexible dosing, low-fill autoinjector

## Abstract

**Background**: Flexibility in dosing volume within an autoinjector platform is critical for streamlining product development, enabling dose adjustments without altering concentration. This study presents a framework to evaluate the capability of a spring-actuated autoinjector platform to deliver fill volumes below its original intended design. **Research design and methods**: This study identifies performance attributes that may be affected by reduced fill volume and introduces a framework to assess them under low-fill conditions. The framework proposes evaluating dose accuracy and injection time using a Zwick machine and analyzing needle dynamics with high-speed imaging. It recommends using SEC, MFI, and HIAC to quantify protein aggregation and subvisible particles for drug product quality assessment. Visual inspection is included to examine syringe integrity. **Results**: The proposed framework was applied to an existing autoinjector platform comprising three models (AI 1, AI 2, and AI 3) designed for nominal fill volumes of 0.5, 1, and 2 mL, respectively, and evaluated at fill-volume reductions of up to 50%. Despite increased driving-rod acceleration and syringe stress under low-fill conditions, all models maintained consistent performance across the evaluated parameters. Dose accuracy was not significantly different from nominal-fill conditions, while injection time decreased by up to 40% at the lowest tested volumes. Peak penetration depth increased significantly for AI 1 at the lowest fill volume (*p* < 0.01); however, final penetration depth remained statistically unchanged for all models. Subvisible particles, primarily attributed to silicone oil droplets, decreased under low-fill conditions. No syringe breakage was observed, and Weibull-based failure probability for AI 3 remained below 10^−7^ across the tested impact-energy range. **Conclusions**: The study confirmed that the framework provides a standardized approach to assess low-fill performance and can inform early platform development and design decisions. This is particularly relevant with the advent of large volume autoinjectors, which may be designed not only to deliver larger doses but also to accommodate smaller fill volumes within the same platform. Overall, this approach supports the development of flexible dosing strategies and may help accelerate timelines from dose selection to regulatory submission.

## 1. Introduction

Single-use, spring-actuated autoinjectors (AIs) are widely adopted for the self-administration of subcutaneous (SC) medications. These devices employ spring-loaded mechanisms to deliver the entire syringe content, often followed by integrated needle-shielding systems post-injection. AIs enable consistent drug delivery, minimize dosing errors and contamination risks, and improve patient safety and usability, contributing to overall reductions in healthcare costs [1,2,3,4].

Platform-based AI development leverages validated, modular components to enable rapid, cost-effective customization for diverse drug products, and is now the prevailing industry approach due to its efficiency, reliability, and reduced time-to-market [5,6,7,8,9,10,11,12,13,14,15,16].

Because dosing regimens are frequently finalized in late-stage development, discrepancies between the selected dose and the AI’s intended delivery volume may arise. This can necessitate inconvenient changes to dosing frequency or modifications to drug concentration, the latter often requiring additional formulation and stability studies. Enabling volume flexibility within an AI platform eliminates the need to adjust drug concentration, thereby streamlining the transition from dose selection to regulatory submission.

**Figure 1:**
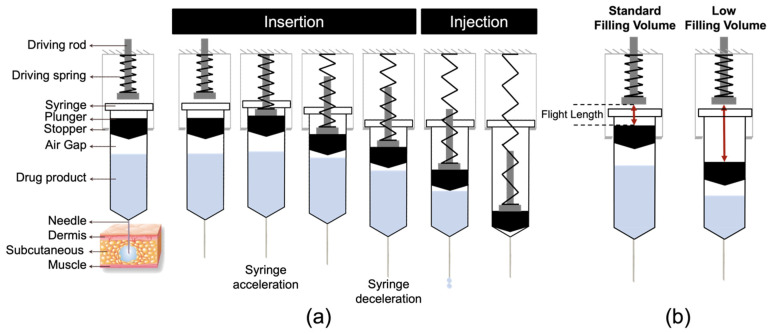
(**a**) The needle insertion and drug injection processes of spring-driven AIs. (**b**) Schematic comparison of plunger position and driving-rod flight length under original design fill volume versus a reduced fill volume, for a constant air-gap height.

Spring-driven AI platforms can be categorized by their activation and insertion mechanisms: push-button and press-body activated. Figure 1a illustrates the working principle of a push-button activated AI platform. In push-button designs, insertion is rapid and fully spring-driven. As shown in Figure 1a, pressing the button causes the syringe to accelerate downward until its flange contacts the stopper and the syringe comes to rest. Injection then occurs as the driving rod advances the plunger to deliver the drug. In contrast, press-body devices require the user to press the device against the skin, initiating a partially manual insertion before the drive spring engages and pushes the plunger to deliver the drug. In both designs, the needle is retracted or shielded upon completion to reduce the risk of needle-stick injuries.

To enable flexible dosing, AIs must deliver volumes lower than their original design specifications while maintaining consistent performance. Without any design change, reducing the fill volume while maintaining a constant air gap between the plunger and drug product results in a lower plunger position within the syringe barrel, as shown in Figure 1b. This increases the flight length between the driving rod and the plunger. From a design and manufacturing standpoint, this is an attractive solution because a single driving-rod configuration can support the delivery of multiple fill volumes. However, the increased flight length allows the spring to accelerate the driving rod over a longer distance, resulting in a higher impact velocity at the plunger. Therefore, the potential effects of this increased impact velocity on device performance are the focus of this study.

Key performance parameters affected by reduced fill volume were identified, including dose accuracy, injection time, syringe robustness, needle dynamics, and drug product quality (subvisible particles).

Dose accuracy [17,18] may be compromised at lower fill volumes because the increased impact velocity of the driving rod on the plunger can induce premature drug dispensing before the needle reaches the target insertion depth. Injection time [17,18] decreases with reduced volume, while injection rate decreases due to lower spring force at the start of delivery.

For designs in which needle insertion is spring-driven, the increased travel distance may increase syringe acceleration and impact velocity when the syringe flange reaches its mechanical stop, which could contribute to flange-region stresses. In addition, increased impact on the plunger can pressurize the syringe contents and transmit stress to the syringe walls. Therefore, syringe integrity remains an important consideration when evaluating low-fill performance across AI configurations.

Additionally, needle dynamics are particularly important in designs where needle insertion is spring-driven. When the syringe flange contacts the stopper at the end of insertion, system compliance can amplify deformation, causing the needle to extend beyond its equilibrium position and increasing insertion depth beyond the design target, which may risk intramuscular rather than subcutaneous delivery [13,19]. Needle extended length is regulated and defined as the distance from the needle tip to the closest patient-contacting surface of the AI [17,18]. Needle lateral movement, though not regulated, was also considered, as consistent needle behavior is desirable [13,19].

**Figure 2:**
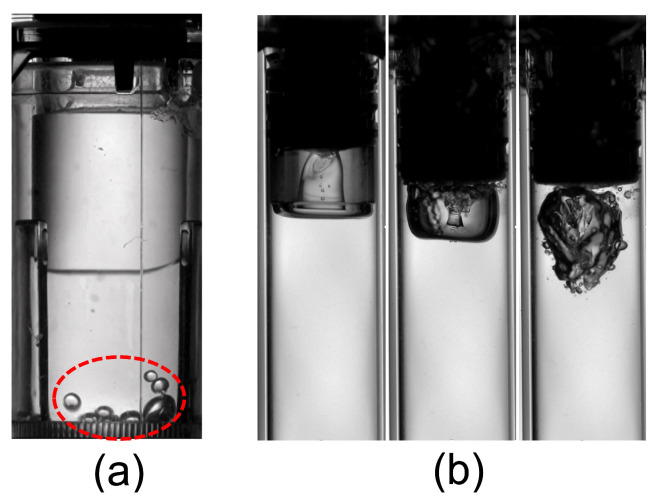
Physical phenomena in the liquid due to syringe acceleration and deceleration. (**a**) Cavitation bubbles, highlighted by the red dashed circle. Reprinted from [20], with permission from Elsevier. (**b**) Liquid sloshing inside the syringe. Reproduced with permission from [26].

Drug product quality and container closure integrity may also be affected. Rapid acceleration and deceleration of syringe in the low-fill condition can lead to increased cavitation (Figure 2a), potentially damaging the syringe and increasing protein aggregation [20,21,22,23,24,25]. Additionally, increased sloshing in the low-fill condition (Figure 2b) may increase interaction with silicone oil or trapped air, risking reduced drug stability [24,25,26].

A methodology framework is proposed to evaluate these effects under low-fill condition. The framework is then applied to a spring driven, push-button AI platform comprising three devices with a different nominal dose volume (0.5 mL, 1 mL, and 2 mL). The results are presented to illustrate the sensitivity of each performance attribute to fill volumes below nominal. The generalizability of the results and the limitations of applying the framework to other platforms are then discussed.

It is worth noting that these individual performance characterization methods, among many others, have previously been reported for AI evaluation. The novelty of this work therefore lies not in introducing new individual test methods, but in integrating these established methods into a targeted framework for assessing whether an existing AI platform can be extended to lower fill volumes without redesigning key mechanical components.

This is particularly relevant as the industry moves toward platform-based design, and as large-volume AIs increasingly enter the market, where a single platform may be expected to support not only higher-volume dosing but also smaller fill volumes to provide greater dosing flexibility.

Although the case study presented here focuses on a push-button AI, the results and discussion of applicability to other designs can inform developers of other AI designs in selecting which performance attributes are most likely to be impacted by reduced fill volumes for their specific platform.

## 2. Methods

As previously outlined, dose accuracy, injection time, needle dynamics, drug product quality, and syringe integrity can be potentially affected under low-fill conditions. This section introduces a systematic framework for evaluating these parameters under low-fill scenarios. Section 2.1, Section 2.2, Section 2.3 and Section 2.4 present generalized evaluation methods that can be customized based on the device design and operating mechanism. Section 2.5 introduces the spring-driven, push-button AI platform used as the case study, for which detailed experimental methods are described in the following subsections.

### 2.1. Methodology to Evaluate Dose Accuracy and Injection Time

Dose accuracy and injection time can be evaluated in alignment with ISO 11608 procedures [17,18]. Under low-fill conditions, premature dispensing may occur before the needle reaches the target insertion depth; therefore, dose accuracy at the intended delivery depth should be captured. To account for this, a collection jar with a septum lid can be used to simulate dermal resistance during device activation [17,18].

### 2.2. Methodology to Evaluate Needle Dynamics

Needle insertion dynamics are particularly important in devices where insertion is spring-driven and can be assessed using high-speed imaging. Higher imaging rates are generally required for spring-driven insertion because the event occurs over a shorter time scale; for example, frame rates of ≥20,000 fps may be appropriate for spring-driven insertion, compared with >500 fps for semi-manual insertion in press-body AIs [13,19]. Devices can be activated using a controlled actuation platform that replicates the user actuation described in the instructions for use while ensuring repeatability. Optical access from two perpendicular planes is recommended to capture the three-dimensional needle trajectory, particularly needle lateral movement. Image post-processing can then be used to track penetration depth and lateral needle displacement throughout insertion.

### 2.3. Methodology to Evaluate Drug Product Quality

Particle generation may arise from both primary-container factors, such as silicone oil amount and distribution, and device-related mechanisms, such as shear, cavitation, air entrainment, and sloshing during activation. Therefore, assessment of subvisible particles under low-fill conditions should isolate the AI-related contributions most likely to be affected by reduced fill volume. For each fill volume, a control sample can be prepared by filling the prefilled syringe to the target volume, allowing representative contact with the syringe barrel, and extracting the solution without AI activation. Comparing this control with the AI-expelled sample helps isolate the contribution of the actuation mechanism to subvisible particle formation and evaluate how this contribution changes across fill volumes.

SEC can be used to assess soluble aggregates and macromolecular degradation, while MFI and HIAC can quantify and characterize subvisible and insoluble particles greater than 2 µm, including differentiation of air bubbles, silicone oil droplets, and protein aggregates.

### 2.4. Methodology to Assess Syringe Failure

To evaluate syringe mechanical integrity under low-fill conditions, visual and magnified inspection should be performed after device activation to identify potential failure locations. In general, reduced fill volume can increase the impact energy transferred to the plunger, pressurizing the syringe contents, and transmitting stress through the syringe barrel. Additional loading conditions may also occur depending on the AI architecture and syringe support within the housing. For example, in spring-driven insertion systems, such as push-button AIs, the syringe may experience higher acceleration during insertion and greater flange impact when it reaches its mechanical stop, making the flange region a critical area for assessment under low-fill conditions. Therefore, beyond visual inspection, syringe strength should be characterized under representative device-specific loading conditions to estimate the probability of syringe failure during low-fill operation.

### 2.5. Case Study: Spring Driven, Push-Button AI Platform

#### 2.5.1. Platform Design

To evaluate the applicability of the proposed framework, we applied it to a spring-actuated, push-button AI platform consisting of three device variants, referred to as AI 1, AI 2, and AI 3. While these models share the same underlying mechanical design, they differ in container closure configurations, delivering nominal fill volumes of 0.5 mL, 1 mL, and 2 mL, respectively.

Table 1 summarizes the needle specifications and drive spring characteristics for each AI model, along with the reduced fill volumes tested to simulate low-fill conditions. The objective of this case study was to determine whether each device could reliably deliver volumes below its original design intent without compromising performance, product quality, or device integrity.

#### 2.5.2. Materials

Two solutions were prepared for this study. Deionized (DI) water (ρ = 998.2 kg/m^3^, μ = 1 cP at room temperature) was used to assess dose accuracy, injection time, and needle dynamics, as its low viscosity has a known impact on these performance attributes.

Prior to syringe filling, the DI water was filtered (0.22 μm) and refrigerated to minimize contamination and preserve fluid properties. In addition, a monoclonal antibody (mAb) solution was used to evaluate the impact of device injection on drug product quality across different fill volumes, with a focus on protein aggregation risk and quantification of subvisible particulate matter. To ensure comparability, all devices within each AI model were filled with the same mAb concentration—90 mg/mL (μ = 3.5 cP) for AI 1 and AI 2, and 150 mg/mL (μ = 5.5 cP) for AI 3.

Syringes were filled using a 10 mL pipette (±0.01 mL accuracy), and plungering was performed using a mechanical vent tube plungering machine (Optima Pharma (Schwäbisch Hall, Germany), Machine No. 4013504). For each fill volume, the machine was calibrated to position the plunger at a defined distance from the syringe flange.

To meet the label claim for each product, an overfill strategy was applied at the standard fill volume, with the target fill set slightly above the label amount. This same overfill and air gap height were applied across lower than nominal fill volumes within each AI model to isolate the effect of fill volume on device performance attributes.

All device components and subassemblies (excluding the filled syringe) were manufactured on qualified production lines at Eli Lilly and Company, representative of commercial-scale processes. Final devices were manually assembled.

#### 2.5.3. Experimental Methodology Applied in Case Study

##### Dose Accuracy and Injection Time

To evaluate dose delivery consistency and timing, 20 devices per case (AI model and fill volume) were tested using a Zwick mechanical tester, following ISO 11608. Each device was activated using a moving rod, with a collection jar positioned beneath the needle. The jar was sealed with a septum lid to simulate dermal resistance and was weighed before activation. After injection, any residual liquid on the septum was removed, and the jar was reweighed. The delivered dose was calculated from the difference between the pre- and post-injection weights. Injection time was identified based on force and position data recorded during the plunger’s travel.

##### Needle Dynamics Characterization

**Figure 3:**
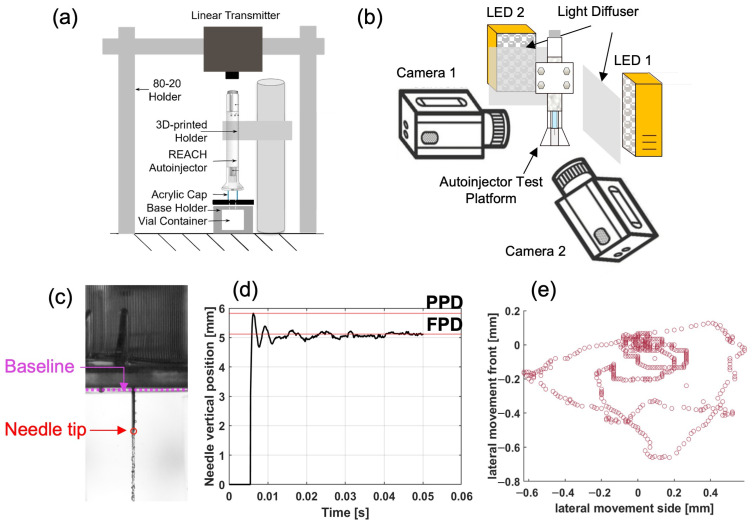
Experimental Setup and Image Processing Workflow for Needle Dynamics Characterization. (**a**) The custom AI test platform secures and remotely activates devices using a motorized actuator. (**b**) Schematic diagram of the experimental setup showing orthogonal high-speed cameras, LED illumination, and positioning fixtures. (**c**) Image processing algorithm identifies the baseline of the injection interface and the needle tip across video frames. (**d**) Needle penetration depth is calculated over time, with Final Penetration Depth (FPD) and Peak Penetration Depth (PPD) indicated. (**e**) Lateral needle motion is extracted from Camera 1 (front view) and Camera 2 (side view), enabling reconstruction of 3D needle displacement trajectories.

High-speed imaging at 20,000 fps and 30 μm/pixel resolution was used to analyze needle motion in 3D. Ten devices per fill volume were mounted on a vibration-dampened fixture and remotely triggered using a linear transmitter moving at 8 mm/s (Fixture Vision Transmitter EB397A1.1 2224). Two synchronized Phantom V2640 cameras captured orthogonal views of needle motion, as depicted in Figure 3a,b. A custom MATLAB (R2023a) algorithm tracked needle tip and device baseline positions to compute penetration depth and lateral movement. This enabled extraction of Peak Penetration Depth (PPD), Final Penetration Depth (FPD), and total lateral displacement using Equation (1), as shown in Figure 3c–e.

(1) $$\begin{array}{c} \mathrm{total}\;lateral\;movement=\sqrt[{2}]{{{lateral\;movement}_{cam\#1}}^{2}+{lateral\;movement_{cam\#2}}^{2}} \end{array}$$

##### Product Quality Evaluation

To evaluate how low-fill conditions impact product quality across AI models, three orthogonal analytical techniques were employed: Size Exclusion Chromatography (SEC), Micro-Flow Imaging (MFI), and HIAC particle counting, in alignment with USP <788> and <789> standards. Each method targeted a different class of particles to provide a comprehensive profile of subvisible and soluble particle formation during the injection process.

For each AI model and fill volume, 6 mL of solution was pooled per condition (e.g., six devices for the 1 mL fill volume, with the number of contributing devices scaling inversely with fill volume for the other conditions). This volume was sufficient to perform 4 replicates of SEC measurements, 2 MFI runs, and 2 replicates of HIAC tests both before and after injection. Because samples were pooled across devices to obtain sufficient volume for the SEC, MFI, and HIAC replicates, this approach does not permit assessment of device-to-device variability in product quality; it instead characterizes the aggregate, condition-level response.

SEC was conducted using a Waters Alliance (Milford, MA, USA) HPLC system with a TSKgel G3000SWXL column to detect soluble aggregates and monomers. This allowed for the quantification of high molecular weight species that might form during shear exposure in low-fill injections.

MFI was performed using a ProteinSimple (San Jose, CA, USA) MFI 5200 system, which combines flow microscopy with image capture to identify and classify subvisible particles ≥2 μm. Shape analysis was used to distinguish:

- Silicone oil droplets (round, smooth edges),
- Proteinaceous aggregates (irregular, jagged edges),
- Air bubbles (high circularity and low contrast).

HIAC 9703+ particle counter, equipped with a 5 mL/min sample sensor, quantified particles ≥2 μm and ≥10 μm, focusing on insoluble particulate matter. All samples were filtered through 1 μm PVDF filters prior to measurement to eliminate dust contamination and ensure reproducibility.

For pre-injection controls, syringes were filled with the target drug product but were not assembled into AIs. The drug solution was carefully withdrawn using a sterile plunger extraction method, ensuring consistent exposure to siliconized syringe barrels.

For post-injection conditions, fully assembled devices were triggered on a Zwick Z010 machine to simulate realistic use. The ejected drug product was collected directly into silicon-free glass vials.

All testing was conducted in a Class 100 laminar flow hood to minimize environmental contamination. This methodology ensured traceable, high-resolution data on the formation and evolution of particles under low-fill injection conditions.

##### Syringe Failure Analysis

To assess the probability of syringe failure under reduced fill volumes, 30 devices per AI model and fill volume were tested. Post-test, all syringes were visually inspected, and five syringes per group were disassembled and examined under magnification to identify signs of structural compromise (e.g., flange cracks or syringe barrel deformation).

For the spring-driven, push-button AI evaluated in this case study, low-fill conditions can increase syringe acceleration during insertion, resulting in higher flange impact when the syringe reaches its mechanical stop. Therefore, the flange region was considered the most critical location for syringe integrity assessment.

A dedicated impact test was performed on syringes outside the AI to represent flange-loading conditions and estimate the probability of flange breakage under different impact loads. This test involved:

- Removing the fluid and fully depressing the plunger, simulating a worst-case configuration.
- Each syringe was mounted in a vertical guide tube fixture, custom-designed to align a controlled-weight impact precisely over the syringe flange.
- A specific weight was dropped incrementally from increasing heights (1-inch increments) until structural failure occurred.
- The drop height was converted to impact energy (N·mm) using gravitational potential energy calculations.
- 20 syringes were tested per case, generating a distribution of failure energies.

**Figure 4:**
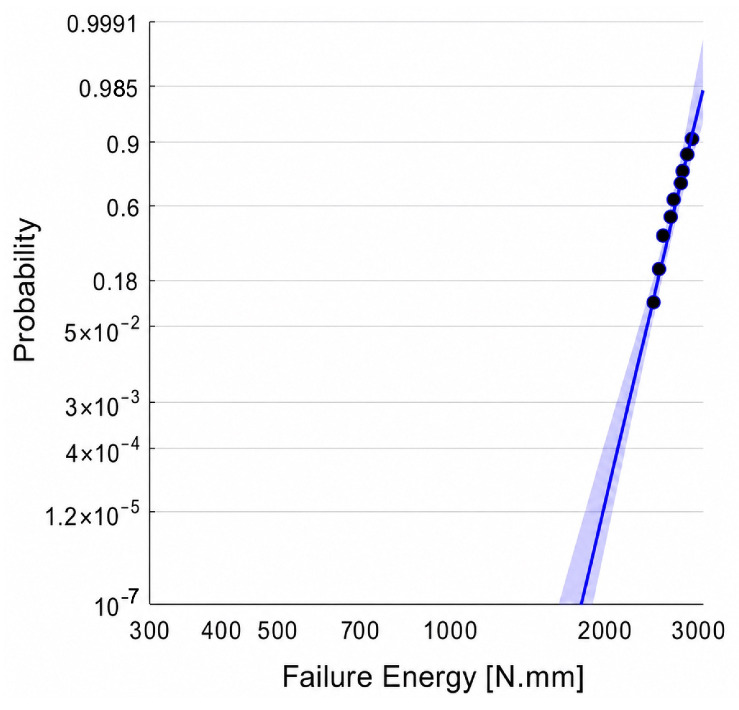
Syringe failure probability as a function of plunger impact energy for syringes used in AI 3. Each point represents the survival outcome of an individual syringe subjected to increasing drop energies in a controlled impact test. A log-normal distribution was fitted to the failure energy data, and the results are plotted on a Weibull scale. The shaded region indicates the confidence band of the fitted distribution.

The resulting data were fitted to a log-normal distribution, and the probability of failure was plotted on a Weibull scale (see Figure 4). This enabled prediction of syringe failure probability at different impact energy levels. For example, syringes used in AI 3 exhibited a six-sigma quality level (3.4 DPMO) at impact energies below 1774 N·mm.

#### 2.5.4. Normalization and Statistical Analysis

For dose accuracy, peak penetration depth, and final penetration depth, results were normalized using the Lower Specification Limit (LSL) and Upper Specification Limit (USL), as shown in Equation (2):

(2) $$\begin{array}{c} \hat{Attribute}=\frac{Attribute-LSL}{USL-LSL} \end{array}$$

This normalization ensures that the normalized LSL and USL correspond to 0 and 1, respectively, across all AI models.

For injection time and maximum lateral movement, each attribute was normalized relative to its median value at the original design fill volume using Equation (3):

(3) $$\begin{array}{c} \hat{Attribute}=\frac{Attribute\;Value}{Median\;Value\;at\;the\;Original\;Design\;Fill\;Volume} \end{array}$$

To illustrate the data distributions, box plots were used. The box represents the interquartile range (IQR), spanning from the first quartile (Q1) to the third quartile (Q3), with a line inside the box indicating the median (Q2). Whiskers extend from the box to the smallest and largest values within 1.5 times the IQR. Points beyond the whiskers are considered outliers [27].

The statistical analysis was performed to compare the distributions of each measured attribute between the low-fill and nominal-fill conditions; it was not intended to estimate population-level confidence bounds for specification compliance. A t-test [27] was conducted to evaluate statistically significant differences in performance between reduced and standard fill volumes. p-values below 0.01 were considered indicative of a statistically significant difference.

## 3. Results

This section presents the results for the case-study platform, a spring-driven, push-button AI comprising three models: AI 1, AI 2, and AI 3, designed to deliver 0.5 mL, 1 mL, and 2 mL, respectively. Each model was evaluated under reduced fill-volume conditions to assess platform robustness. The following subsections characterize the platform’s performance under reduced fill volume, covering mechanical behavior (dose accuracy, injection time, and needle dynamics), container closure integrity, and drug product quality attributes.

### 3.1. Dose Accuracy

**Figure 5:**
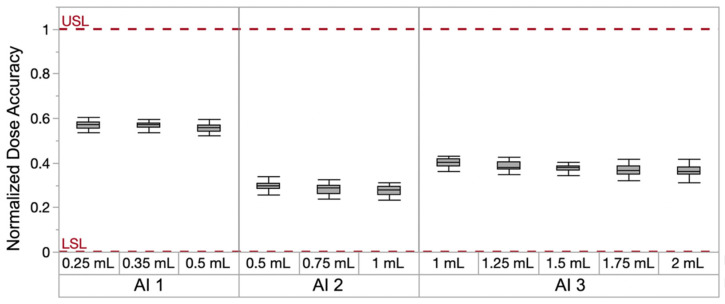
Normalized dose accuracy for different AI models (AI 1, AI 2, and AI 3) and various fill volumes, presented as box plots. Within each device, dose accuracy is normalized using the upper and lower specification limits (USL and LSL), represented by red dashed lines set at 1 and 0, respectively.

Figure 5 shows the distribution of normalized dose accuracy for the three AI models (AI 1, AI 2, and AI 3) under nominal and low-fill conditions. Across all models and fill volumes, normalized dose accuracy remained within the specification range of 0 to 1, corresponding to the lower and upper specification limits (LSL and USL). No statistically significant differences were observed between nominal and low-fill conditions.

To further assess dose accuracy, tolerance intervals were calculated with a confidence level of 0.95 and proportion of 0.99, knowing the attribute is normally distributed and independent. The results, shown in Table 2, demonstrate that the upper and lower tolerance intervals remain within the specification limits (0–1) for all test groups. This indicates that, with 95% confidence, at least 99% of the devices deliver doses within the specified range, even when they are filled to deliver a volume that is less than the initial design intent.

### 3.2. Injection Time

**Figure 6:**
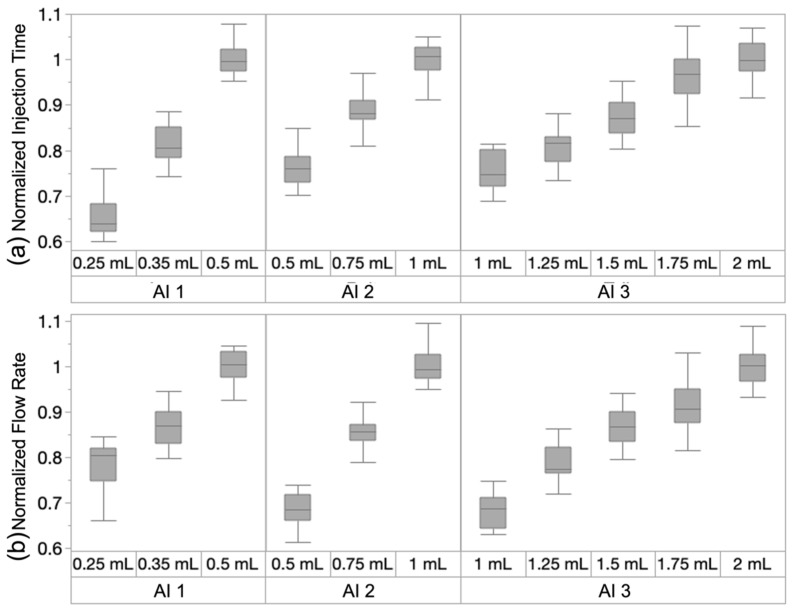
(**a**) Normalized injection time and (**b**) Normalized flow rate distributions across AI models and fill volumes.

Figure 6a presents the distribution of normalized injection times for three AI models under original design and low-fill conditions, shown as box plots. The data demonstrate that decreasing the fill volume results in shorter injection times across all AI models. To further evaluate the impact of reduced injection times, the average flow rate was calculated as the ratio of delivered dose to injection time, and the results are shown in Figure 6b. As hypothesized, the flow rate decreases under low-fill conditions across all AI models. This occurs because, with a lower fill volume, the drive spring remains less compressed throughout the injection, resulting in reduced energy release and, consequently, lower energy transfer to the liquid compared to a full-fill condition.

### 3.3. Needle Dynamics

**Figure 7:**
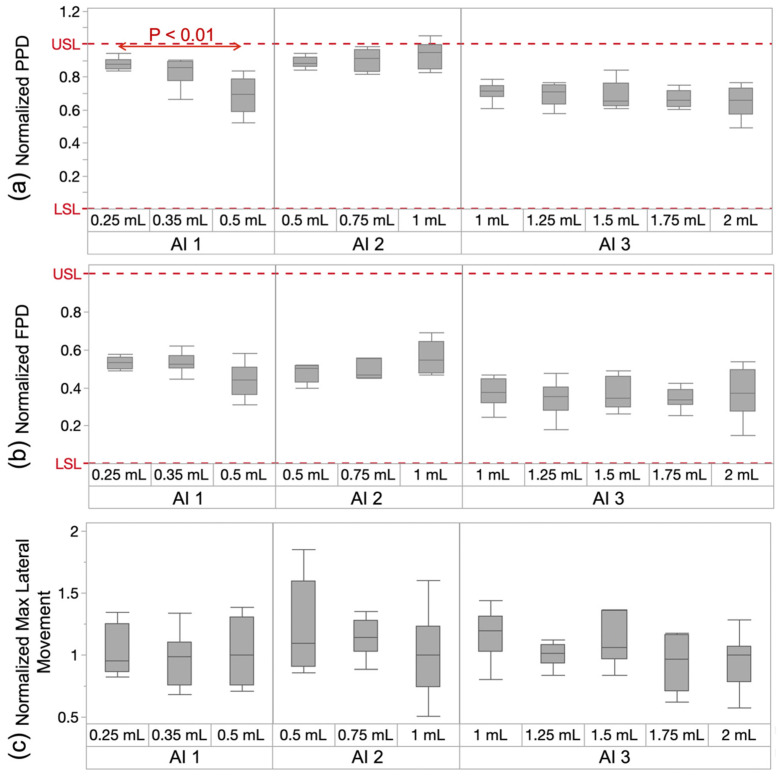
(**a**) Normalized Peak Penetration Depth (PPD), (**b**) Normalized Final Penetration Depth (FPD), and (**c**) Normalized Maximum Lateral Movement distributions for different devices (AI 1, AI 2, and AI 3) across various fill volumes, presented as box plots. Significant differences are indicated by red *p*-values within the plots.

Figure 7a–c show the distribution of normalized needle peak penetration depth (PPD), final penetration depth (FPD), and maximum lateral movement, respectively, across different AI models and filling volumes. While there is no specification limit for PPD, both PPD and FPD are normalized with the upper and lower specification limits (USL and LSL) for FPD to facilitate comparison. Across all AI models and filling volumes, PPD values consistently exceed FPD values. As shown in Figure 7a, PPD distribution remains consistent between the original and lower fill volumes for AI 2 and AI 3. However, for AI 1, a statistically significant difference (*p*-value < 0.01) is observed in the PPD distribution between the original design volume (0.5 mL) and the lowest filling volume (0.25 mL), suggesting a possible dependency on device design that might increase the risk of intramuscular injection. Despite significant PPD variation in AI 1, Figure 7b shows that FPD remains consistent across all fill volumes for all three AI models, with no significant differences between the original design and lower fill volumes. Since delivery occurs at the final rather than peak depth, this PPD shift is not expected to meaningfully affect the delivery route, and the device continues to meet its subcutaneous delivery intent at reduced fill volumes.

Figure 7c shows no significant difference in the distribution of maximum lateral movement across fill volumes for all AI models, suggesting that fill volume does not affect lateral movement in this platform design.

### 3.4. Product Quality

**Figure 8:**
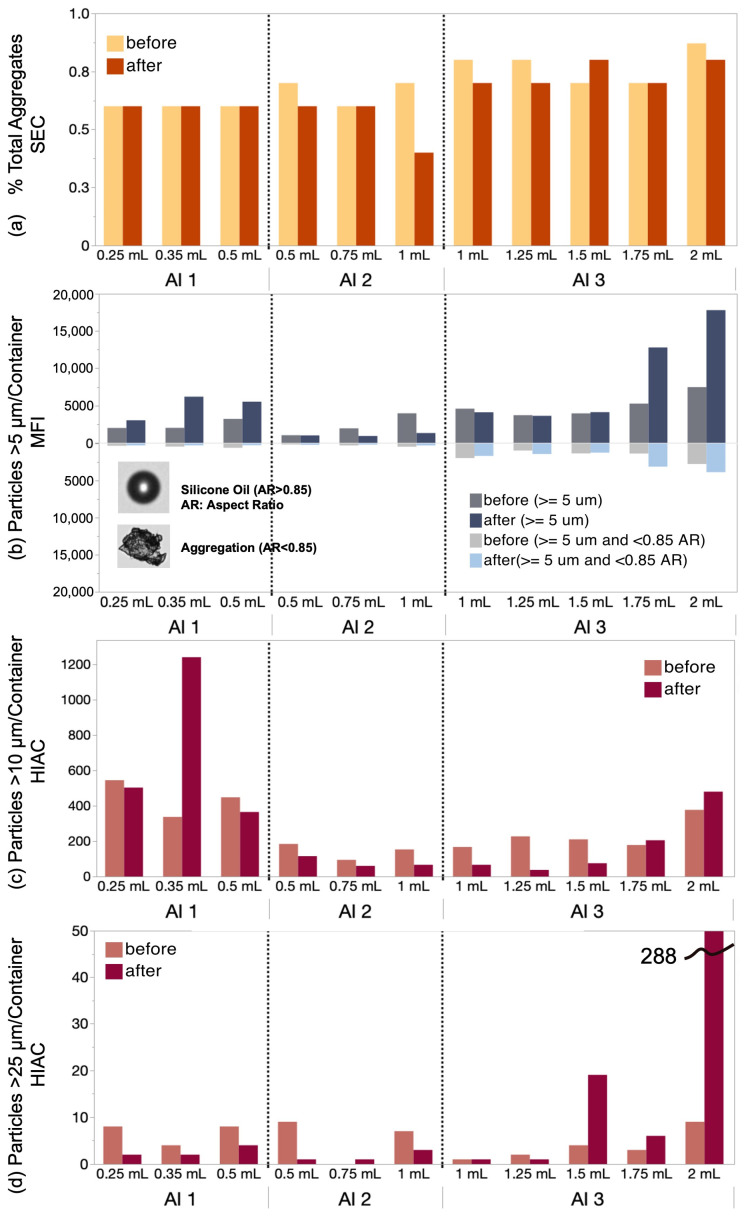
(**a**) Percentage of total aggregates observed with SEC, with yellow bars showing pre-injection values and orange bars showing post-injection values. (**b**) MFI particle counts for particles larger than 5 µm per container, with dark gray and blue bars representing total particle counts before and after injection, respectively, and light gray and blue bars representing non-circular particles (aspect ratio < 0.85). (**c**,**d**) HIAC measurements for particles larger than 10 µm and 25 µm per container, with light pink bars showing pre-injection data and dark pink bars showing post-injection data.

Figure 8 illustrates the number of soluble and insoluble particles in the drug solution before and after injection across different AI models and fill volumes. Since the same formulation was used within each AI model, the reported differences reflect the effects of device and fill-volume conditions only.

Figure 8a compares the percentage of soluble aggregates (i.e., non-monomer species) before injection (yellow bars) and after injection (orange bars). In all cases, aggregates accounted for less than 1% of total protein content, with no significant differences observed between fill volumes or injection states. These results suggest that fill volume does not affect protein aggregation under the tested conditions.

Figure 8b presents MFI results for particles larger than 5 µm. Dark gray and blue bars represent the total number of particles per container before and after injection, respectively, while light gray and light blue bars indicate the number of non-circular particles (defined as having an aspect ratio <0.85). The data show that the majority of subvisible particles were circular, implying that they were primarily silicone oil droplets rather than proteinaceous aggregates. Silicone oil is used as a syringe lubricant and may enter the drug solution either due to plunger movement during delivery (which causes friction along the syringe barrel) or from increased fluid mixing induced by sloshing when the syringe accelerates.

Figure 8c,d display HIAC measurements of particles greater than 10 µm and 25 µm, respectively. These size thresholds are of regulatory interest due to specified limits on the number of particles per container. Light pink bars represent pre-injection measurements, while dark pink bars show post-injection results. The HIAC data were consistent with MFI trends, indicating a general reduction in particle counts at reduced fill volumes compared to standard label claim volumes. An exception was observed for AI 1, where the number of particles ≥10 µm increased at lower fill volumes.

Across all subplots, a slight post-injection reduction in particle counts was occasionally observed. This is attributable to inherent measurement variability and not considered physically meaningful, as both MFI and HIAC methods have high variability, with Relative Standard Deviations (RSD) exceeding 30%.

### 3.5. Syringe Failure

Visual inspection confirmed the absence of cracks or structural breakages in all syringes following injection for AI 1, 2, and 3. As a showcase, a Weibull analysis was conducted for AI 3 devices using the methodology described in Section 2.5.3. The resulting Weibull plot demonstrated that the probability of syringe failure remained below 10^−7^, even at elevated plunger impact energies. These findings underscore the mechanical robustness of the syringe system across varying fill volumes and operational conditions.

## 4. Discussion

AIs are designed to deliver a precise subcutaneous dose, typically requiring the drug product volume to match the device’s intended fill volume. When this alignment is not feasible, formulation changes, such as concentration adjustment, may be required, which can trigger additional stability studies and extend development timelines. Enabling a single AI platform to reliably deliver a range of fill volumes without design changes can support flexible dosing and accelerate development. This is increasingly relevant as the industry moves toward platform-based development and large-volume AIs that may also need to accommodate smaller doses.

This study presents a framework to evaluate AI performance under low-fill conditions without altering device components. Under low-fill conditions, the plunger sits lower in the syringe barrel, increasing the travel distance of the driving rod before impact and potentially increasing impact energy on the plunger.

This work focuses on identifying which AI performance attributes may be adversely affected by this increased impact energy and what test-method considerations are particularly important under low-fill conditions. Based on AI performance requirements and relevant standards, including ISO 11608 and USP <788>/<789>, we hypothesize that dose accuracy, injection time, needle dynamics, syringe robustness, and subvisible particle formation may be affected by low-fill operation. Although these attributes are commonly evaluated separately, this work integrates them into a targeted assessment of low-fill feasibility and highlights key test-method considerations.

The framework was applied to a push-button, spring-actuated AI platform as a case study. The results are discussed in the following sections, along with the applicability of the framework to other AI designs and potential mitigation strategies if performance requirements are not met.

### 4.1. Dose Accuracy and Delivery of the Label Claim

Dose accuracy is a critical performance attribute for delivery systems because it directly affects the delivered therapeutic dose and, consequently, therapeutic efficacy. Under low-fill conditions, increased impact energy on the plunger may compress the air gap in the syringe more forcefully, increasing the risk of premature drug dispensing before the needle reaches the intended insertion depth, particularly for low-viscosity solutions.

Within the proposed framework, testing with water at room temperature is recommended as a conservative condition for low-viscosity formulations. Because premature dispensing may occur before the needle reaches the target insertion depth, dose accuracy should be assessed at the intended delivery depth. To capture this effect, a collection jar with a septum lid can be used to simulate dermal resistance during device insertion.

In the case study, no significant change in dose accuracy was observed under low-fill conditions compared with nominal fill volume, and all models maintained acceptable dose accuracy. However, premature dispensing may depend on spring configuration, activation mechanism, air-gap size, and formulation viscosity. Therefore, these dose-accuracy results should not be directly generalized to other designs; instead, this attribute should be evaluated using the proposed methodology for each platform.

If dose accuracy cannot be maintained, potential mitigation strategies include using a longer driving rod to reduce free-flight distance or adjusting the filling strategy to increase overfill for low-volume products.

### 4.2. Needle Dynamics (PPD, FPD, and Lateral Movement)

Accurate subcutaneous delivery depends on controlled needle insertion depth. In this framework, needle depth is characterized using Peak Penetration Depth (PPD) and Final Penetration Depth (FPD). Under low-fill conditions, increased impact energy may transiently drive the syringe and needle deeper, potentially increasing PPD and, in some cases, FPD. A higher PPD may increase the risk of momentary intramuscular penetration, which could contribute to pain due to the higher density of nerve endings in muscle tissue [28,29,30]. Although FPD is typically more relevant to final drug deposition and absorption [31,32], both PPD and FPD are important for consistent delivery.

The effect of low-fill operation on needle depth depends on device architecture, including the syringe housing material, geometry, and mechanical constraints. Elastic housing deformation may temporarily increase PPD under impact; however, if the housing returns to its original shape and no permanent deformation occurs, FPD is expected to remain primarily governed by the final syringe position. This behavior was observed in the case study. One model showed a significant increase in PPD under low-fill conditions, while FPD remained unchanged across all models compared with nominal fill volume. This suggests that transient increases in needle depth may occur depending on spring configuration, impact severity, and housing compliance, whereas changes in FPD are less likely unless permanent deformation or altered final syringe positioning occurs. The risk of transient needle-depth increase is expected to be more relevant for spring-driven insertion systems than for designs with manual or semi-manual insertion.

Although needle lateral movement is not defined as an Essential Drug Delivery Output (EDDO), it may affect patient comfort and consistency of needle behavior. Under low-fill conditions, increased impact energy may increase lateral needle motion depending on the insertion mechanism and syringe constraints. In the case study, lateral movement did not change significantly under low-fill conditions; however, this response may vary across platforms depending on device architecture and mechanical support of the syringe.

### 4.3. Injection Time and Flow Rate

Injection time is an important performance attribute because excessively long delivery times may increase the risk of premature device removal by the user. At lower fill volumes, injection time typically decreases because a smaller volume is delivered, which may improve user compliance. In compression-spring-driven systems, the injection flow rate may also decrease because the spring force at the start of delivery is lower under low-fill conditions. Consistent with this expectation, both injection time and flow rate decreased with reduced fill volume in the case study.

This trend is expected for devices that use a compression spring as the drive unit. In contrast, devices with a constant-force or torsional spring may maintain a more consistent flow rate across fill volumes. In either case, reduced fill volume is not expected to introduce a performance risk related to injection time and injection flow rate.

### 4.4. Drug Product Quality

Per USP <788>/<789>, subvisible particle (SVP) content per container is subject to defined limits, making control of these effects critical for product quality. Under low-fill conditions, the reduced drug volume results in less contact between the formulation and the syringe wall and therefore less exposure to silicone oil, used as a syringe lubricant. However, we hypothesize that SVP levels may nonetheless increase due to the higher plunger impact energy. Increased impact on the syringe plunger may intensify sloshing and cavitation, generating high shear forces within the drug solution [20,21,22,23,24,25,26]. These effects can lead to protein aggregation or incorporation of air bubbles and silicone oil into the formulation.

Although sloshing occurs rapidly (with exposure to high shear rates lasting ~50 ms) [26], it increases the air–liquid interface, posing a risk that can intensify protein aggregation. Cavitation risk increases with higher impact on plunger; however, the reduced liquid height in low-fill conditions mitigates this to some extent. Analytical techniques such as Size-Exclusion Chromatography (SEC), Micro-Flow Imaging (MFI), and HIAC particle counting are recommended to verify product quality under these conditions.

In our case study, protein aggregation was minimal under low-fill conditions. SEC analysis showed aggregate levels remained below 1% pre- and post-injection. MFI analysis revealed that most subvisible particles >2 μm were circular, suggesting they were silicone oil droplets rather than protein aggregates.

Formulation sensitivity to shear varies between drug products; therefore, the product quality findings reported here, specifically the absence of protein aggregation concern, cannot be generalized to all formulations or platform designs. Product quality under low-fill conditions should be evaluated independently for each combination of formulation and AI platform.

### 4.5. Syringe Integrity and Failure Risk

Maintaining syringe structural integrity is critical for safety and performance. In general, reduced fill volume increases the impact energy transferred to the plunger, pressurizing the syringe contents, and transmitting stress through the syringe barrel. Additional loading conditions may arise depending on the AI architecture and how the syringe is supported within the housing. For example, in spring-driven insertion systems such as push-button AIs, the syringe may experience higher acceleration during insertion and greater flange impact upon reaching its mechanical stop, making the flange region a critical area for assessment under low-fill conditions.

In the present case study, which features a spring-driven insertion, Weibull analysis was used to estimate the probability of syringe failure at the flange region under representative loading conditions. Results demonstrated negligible failure probabilities for AI 3 across all tested fill volumes, indicating robust mechanical reliability under low-fill operation.

For other platforms, visual inspection alone is insufficient; syringe strength should be characterized under device-specific loading conditions representative of low-fill operation. Failure probability analysis using Weibull distribution modeling can quantify reliability and inform material selection or design modifications. Should the estimated failure rate exceed six-sigma quality thresholds (3.4 defects per million), alternative materials or structural reinforcements—such as changes to the syringe barrel or carrier design—may be warranted.

## 5. Conclusions

This study introduces a systematic framework to evaluate AI performance under low-fill conditions, scenarios where fill volumes are reduced below the original design intent without any hardware modifications. The framework identifies critical performance attributes at risk, including dose accuracy, injection time, needle dynamics, drug product quality, and syringe integrity.

The proposed framework offers a standardized methodology to identify and test key performance parameters under low-fill scenarios, and can serve as a design tool for emerging AI platforms. If performance deficiencies are observed, adjustments in design (e.g., driving rod length) or fill strategies can be implemented early in development, improving robustness and accelerating time-to-market.

These test methods were applied to a case study comprising three spring-actuated, push-button AI models designed to deliver 0.5 mL, 1 mL, and 2 mL doses, respectively. Consistent and reliable performance was observed across reduced fill volumes. Despite theoretical risks associated with low-fill operation, including increased plunger velocity, higher impact forces, and potential changes in needle dynamics, the experimental results showed no compromise in key functional performance attributes. Analytical methods (SEC, MFI, HIAC) confirmed minimal protein aggregation and low subvisible particulate formation, while visual inspections and probabilistic failure modeling confirmed syringe integrity remained within acceptable safety margins.

## Figures and Tables

**Table 1:** Test Matrix, along with needle and drive spring characteristics for each device.

Device	Original Design Volume [mL]	Nominal Air Gap Height at Original Design Volume [mm]	Tested Volumes [mL]	Needle Gauge	Needle Length [mm]	Spring Stiffness [N/mm]
AI 1	0.5	5	0.25, 0.35, 0.50	29 G	12.7	0.22
AI 2	1.0	5	0.50, 0.75, 1.00	27 G	12.7	0.20
AI 3	2.0	5	1.00, 1.25, 1.50, 1.75, 2.00	27 G	8.0	0.26

**Table 2:** Upper and lower tolerance intervals (TIs). The TI values are calculated for normalized dose accuracy (DA) and, as shown in the table, fall within the DA normalized upper specification limit (USL) and lower specification limit (LSL).

	AI 1			AI 2			AI 3				
Tested Volume (mL)	0.25	0.35	0.50	0.50	0.75	1.00	1.00	1.25	1.50	1.75	2.00
USL	1.00	1.00	1.00	1.00	1.00	1.00	1.00	1.00	1.00	1.00	1.00
Upper TI	0.68	0.61	0.64	0.34	0.41	0.32	0.45	0.44	0.41	0.43	0.42
Lower TI	0.47	0.52	0.46	0.25	0.17	0.23	0.36	0.32	0.34	0.30	0.30
LSL	0.00	0.00	0.00	0.00	0.00	0.00	0.00	0.00	0.00	0.00	0.00

## Data Availability

Data Availability Statement: The data supporting the findings of this study are presented within the article. The raw data are not publicly available due to confidentiality and proprietary restrictions. Further inquiries can be directed to the corresponding author.
